# Viscosity affected by nanoparticle aggregation in Al_2_O_3_-water nanofluids

**DOI:** 10.1186/1556-276X-6-248

**Published:** 2011-03-22

**Authors:** Fei Duan, Dingtian Kwek, Alexandru Crivoi

**Affiliations:** 1 School of Mechanical and Aerospace Engineering, Nanyang Technological University, Singapore 639798, Singapore

## Abstract

An investigation on viscosity was conducted 2 weeks after the Al_2_O_3_-water nanofluids having dispersants were prepared at the volume concentration of 1-5%. The shear stress was observed with a non-Newtonian behavior. On further ultrasonic agitation treatment, the nanofluids resumed as a Newtonian fluids. The relative viscosity increases as the volume concentrations increases. At 5% volume concentration, an increment was about 60% in the re-ultrasonication nanofluids in comparison with the base fluid. The microstructure analysis indicates that a higher nanoparticle aggregation had been observed in the nanofluids before re-ultrasonication.

## Introduction

Nanofluids, consisting of solid nanoparticles at about 1-100 nm, have drawn greater attention since they are expected to exhibit superior properties compared with conventional heat transfer fluids [1-3]. Nanoparticles which have a much larger surface area and smaller size possess a potential to further improve heat-transfer capabilities and increase the stability in the fluids. Nanofluids would have a lower viscosity than the conventional micron-sized particle-liquid suspensions, thus reducing pressure drop in the flow channel and saving the pumping power. The experiments on the nanofluid viscosity [4,5] demonstrated up to 90% increment in a 5% volume fraction nanofluid compared with the base liquid. The result was far higher than the theoretical prediction from Einstein, Brinkman, and Batchelor models [6-11]. In addition, most reported data on the thermal properties seem to be measured in the fresh well-dispersed nanofluids. A further understanding of nanofluid stability is necessary before nanofluids can be commercialized in the practical applications. To improve the stability of nanofluids, mixing of dispersants [12,13], surface treatment of nanoparticles [14], and ultrasonication treatment [15] have been used to minimize particle aggregation in the base fluids. However, Das et al. indicated an increase of viscosity with increased particle concentrations in Al_2_O_3_-water nanofluids [16]. The possibility of non-Newtonian fluids might be found in the higher concentration nanofluids, where the nanoparticles could aggregate. Pastoriza-Gallego et al. indicated that the differences in size or aggregation of the nanoparticles have a determining influence on the viscosity of nanofluids [17]. However, few studies have systemically addressed the effect of the nanoparticle aggradation on the viscosity in the nanofluids. Thus, the viscosity variation of Al_2_O_3_-water nanofluids kept 2 weeks between before and after re-ultrasonication treatment is investigated in this article.

## Experimental

In the nanofluid preparation, we dispersed the Al_2_O_3 _nanoparticles with an average diameter of 25 nm and a particle density of 3.7 g/cm^3 ^(Nanostructured and Amorphous Materials) into 100 mL of the deionized water to make up the volume concentrations from 1 to 5% with an interval at 1%. Additional 0.01 vol% surfactant, cetyltrimethy-lammonium bromide, was mixed in the nanofluids [12,13]. Then, the suspension was stirred on a magnetic plate before subjecting to ultrasonication process (Fisher Scientific Model 500). The purpose of mixing of dispersants and ultrasonication treatment is to ensure uniform dispersion of nanoparticles as well as to prevent the nanoparticles from the initial agglomerating in the base fluid. The viscosities of nanofluid were measured 2 weeks after they had been prepared. Thereafter, the nanofluids were measured again just after re-ultrasonication.

In both the above conditions, the viscosity of Al_2_O_3_-water nanofluids were measured using a controlled rate rheometer (Contraves LS 40) which has a cup-and-bob geometry. The bob is connected to the spindle drive while the cup is mounted onto the rheometer. As the cup is rotated, the viscous drag of the fluid against the spindle is measured by the detection of the torsion wire. The cup-and-bob geometry requires only a sample volume of approximately 5 mL. Satisfactory results were produced when the applied torque was between 10 and 100% of the maximum permissible torque. Hence, during measurements, the readings were discarded if the applied torque did not fall within this prescribed range. The experimental apparatus was calibrated by measuring the viscosity of the deionized water. Based on the calibration results, the measurement error was controlled within ± 1%. All the measurements were conducted at the atmospheric pressure and the room temperature. To differentiate the particle distribution in the nanofluids, a small droplet was sampled from the 5 vol% nanofluid, held for 2 weeks and after re-ultrasonication respectively; then, it was dried on a clean polymethyl methacrylate plate. The dried samples were coated with Au for observing the morphology of the crystallization under a scanning electron microscopy (SEM, Jeol).

## Results and discussion

**Figure 1:**
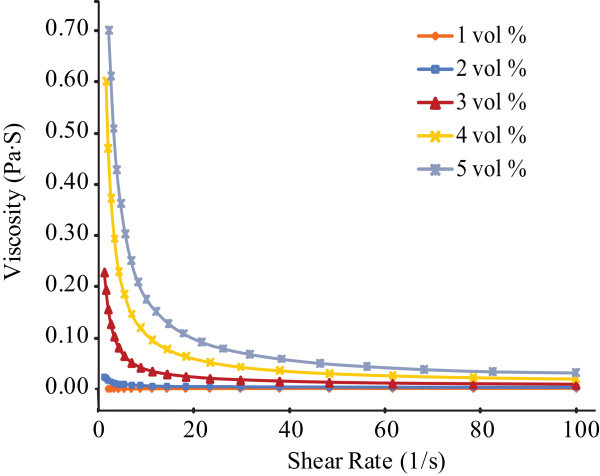
**Viscosity as a function of shear rate in Al_2_O_3_-water nanofluids at the volume concentration from 1 to 5% (after 2 weeks)**.

**Figure 2:**
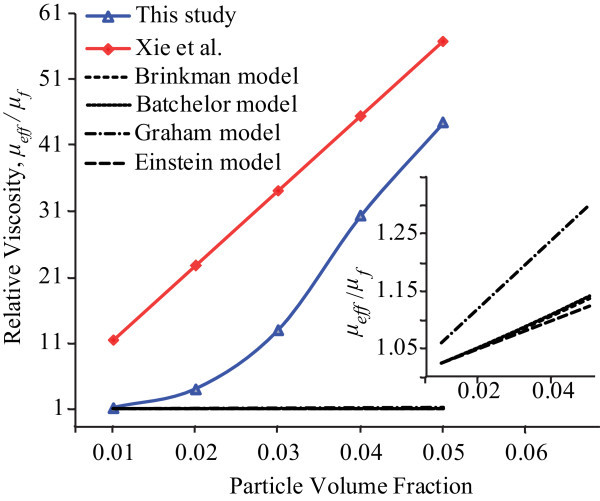
**Relative viscosity of Al_2_O_3_-water nanofluids as a function of volume con-centration (after 2 weeks)**.

The viscosity measurement was taken 2 weeks after the nanofluid preparation. As seen in Figure 1, the viscosity decreases as the shear rate increases. At a certain shear rate, the nanofluid at 5 vol% has the largest viscosity while the viscosity value is the lowest in the 1 vol% nanofluid. The nanofluids behaved as non-Newtonian fluids. The effective viscosity, *μ*_eff_, of nanofluids increases up to about 38 × 10^-3 ^Pa·S for the 5 vol% nanofluid. Figure 2 shows that the relative viscosity, *μ*_eff_/*μ*_f _(*μ*_f _is the viscosity of the base fluid) increases from the above value for the 1 vol% nanofluid to about 43 for the 5 vol% nanofluid. However, the values are much higher than the those predicted from the conventional Einstein model, and those of the modified models by Brinkman, Batchelor, and Graham [6,7,10,11]. The data of Xie et al. [18] show a similar phenomenon also as shown in Figure 2. The nanoparticles were indicated to be prone to form agglomeration in a nanofluid suspension. The high viscosity observed is probably as a result of agglomeration that had occurred in the nanofluids after 2 weeks. Once agglomeration is formed, a larger stress is necessary to break the ligand structure among particles when shearing takes place; therefore, a high relative viscosity would be observed in the fluids as shown in Figures 1 and 2. Zhou et al. [19] also highlighted that the shear thinning behavior at high shear rate is likely due to aggregates being destroyed under shear. This can also explain that the non-Newtonian characteristics of nanofluids are more obvious at a higher volume fraction and a longer holding time since the chance of aggregation is higher. The aggregates are also verified in the following SEM images.

**Figure 3:**
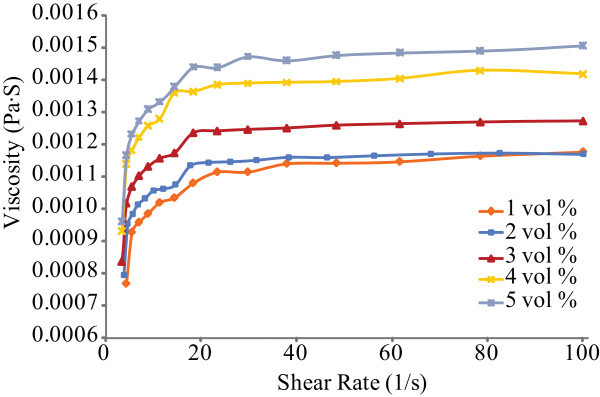
**Viscosity as a function of shear rate for Al_2_O_3 _nanofluids at the volume concentrations from 1 to 5% (after re-ultrasonication)**.

**Figure 4:**
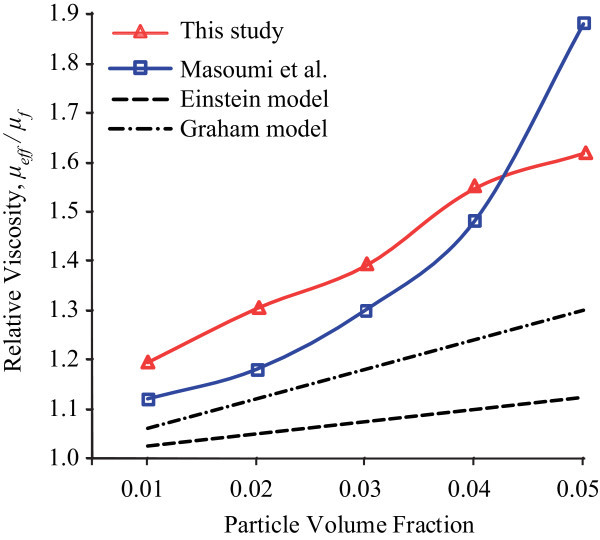
**Relative viscosity of Al_2_O_3 _nanofluids as a function of volume concentration (after re-ultrasonication)**.

Re-ultrasonication process was conducted on the 2-week Al_2_O_3_-water nanofluids in order to disperse the aggregated nanoparticles before the viscosity was measured again. Figure 3 demonstrates that the viscosity increases with the shear rate roughly linearly at the beginning before it reaches a constant value for each fluid. The nanofluids resume Newtonian. The nanofluid at 5 vol% has the largest viscosity while the value is the lowest in the 1 vol% nanofluid. Distinctively, it is seen that the relative viscosity is much lower than the relative nanofluid before re-ultrasonication, as illustrated in Figure 4. After re-ultrasonication, the effective viscosity gets back the values in the freshly prepared nanofluids [20]. The relative viscosity increases as the volume concentration increases. It supports the hypothesis that a high viscosity might be due to nanoparticle agglomeration. The results reported by Masoumi et al. [4] show a similar trend, too. From these experimental results, the measured relative viscosity of Al_2_O_3_-water nanofluids is significantly 60% higher than those of the base fluid in the nanofluids after the non-Newtonian fluids were ultrasonically agitated again. The measures of Masoumi et al. and this research are much higher than those of the predicted values given by the Einstein and Graham equations [6,11]. Clearly, the Einstein formula and the others have underestimated the nanofluid viscosities [6-11]. For higher particle concentrations, the deviation of conventional models from the present experimental data is considerable. Even the Batchelor formula that considers the Brownian effects performs poorly [10]. Chandrasekar et al. [21] suggested that the significant difference between the experimental results and the predicted values might be because of the conventional models neglecting the hydrodynamic interactions between particles which become important, as the other disturbances of the fluid around one particle might interact with the surrounding particles at higher volume concentrations. The nanoparticle aggregation in the fluids would reinforce the effects.

**Figure 5:**
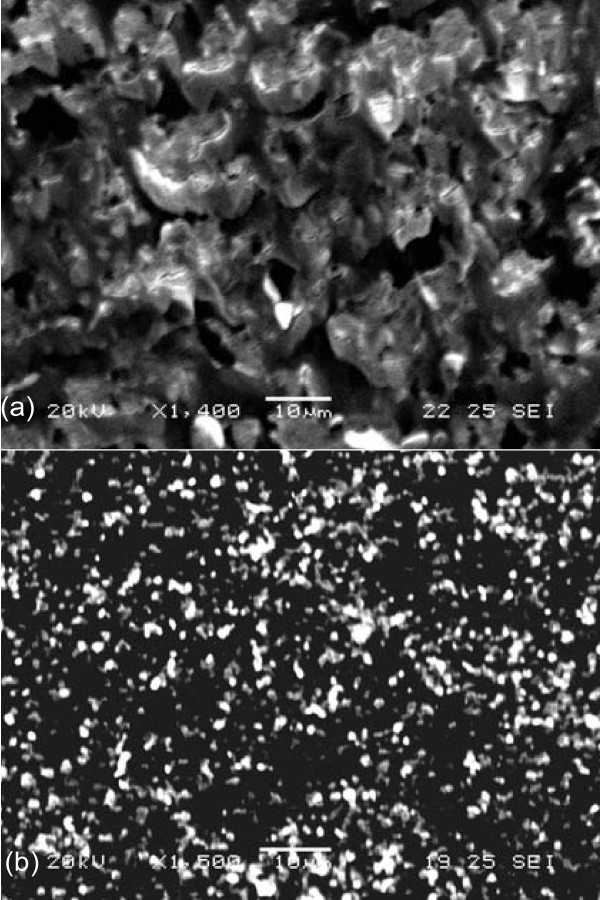
**SEM microstructure of the dried Al_2_O_3_-water nanofluids before re-ultrasonication **(a) **and after re-ultrasonication (b)**.

As illustrated in Figure 5, the microstructure of the nanoparticle distribution was measured after sampling and drying the drops at 5 vol% nanofluids which were held for 2 weeks and after re-ultrasonication. The nanoparticles accumulated together in a micron scale before re-ultrasonication of the 2-week nanofluids as seen in Figure 5a, while the slight aggregation of nanoparticles was well dispersed after re-ultrasonication as seen in Figure 5b.

As suggested by Chen et al. [22], the nanoparticles in the fluid are likely to form aggregates. We can apply the Krieger and Dougherty model to explain the relative viscosity, *μ*_eff/_*μ*_f_, qualitatively [23](1)

where [*η*] is the intrinsic viscosity with a value of 2.5 for hard spherical particles, *ϕ_m _*is the volume fraction of densely packed spheres, *ϕ*_a _is the volume fraction of aggregates, expressed as , *d*_a _is the diameter of aggregates, *d *is the nominal diameter of particle, *d*_f _is the fractal dimension of the aggregates, and *ϕ *is the volume fraction of the well-dispersed individual particles. If there is no agglomeration, then Krieger and Dougherty model can be reduced to the ideal Einstein model [6]. However, it is impossible to eliminate the agglomeration in nanofluids completely. Thus, the magnitude of *d*_a_*/d *in the nanofluids is larger than 1. As the size of the aggregates increases, the relative viscosity will increase. In addition, as the shape of the aggregate is no longer spherical due to aggregation, the intrinsic viscosity should be greater than 2.5 for other shapes [24]. This can also account for the increase in the viscosity as the nanoparticle aggregate size is larger in the 2-week nanofluids before re-ultrasonication than that after re-ultrasonication. It might also partially explain as to show a higher concentration nanofluid has a larger relative viscosity because the 5 vol% nanofluid has a higher possibility for forming agglomerates in comparison with the 1 vol% nanofluid.

## Conclusion

Viscosity measurement shows that the 2-week Al_2_O_3_-water nanofluids at the volume concentration of 1-5% are not Newtonian as seen in Figure 1. The relative viscosity is much higher than that in the nanofluids after re-ultrasonication (Figures 2 and 4). The re-ultrasonication treatment resumed the nanofluids as a Newtonian fluid. The relative viscosity increases up to about 60% in comparison of the base fluid as the volume concentrations increase to 5 vol%. The huge deviation between the experimental results and those of the present theoretical models might be due to the nanoparticle agglomeration (Figure 5). It will be imperative to conduct more detailed studies of particle agglomeration in the nanofluids and the effects on the thermal properties to stabilize nanofluid for applications in the near future.

## Abbreviations

SEM: scanning electron microscopy.

## Competing interests

The authors declare that they have no competing interests.

## Authors' contributions

DK carried out the experimental studies and participated in drafting the manuscript. AC participated in analyzing of the data. FD conceived of the study, and revised the manuscript. All authors approved the final manuscript.
